# Inferring biological kinship in ancient datasets: comparing the response of ancient DNA-specific software packages to low coverage data

**DOI:** 10.1186/s12864-023-09198-4

**Published:** 2023-03-14

**Authors:** William A Marsh, Selina Brace, Ian Barnes

**Affiliations:** 1 Natural History Museum, Cromwell Road, SW7 5BD London, England; 2 BioArCh, University of York, YO10 5NG York, England

**Keywords:** Ancient DNA, Biological kinship, Relatedness, Low coverage, Pseudohaploid, Genotype likelihood

## Abstract

**Background:**

The inference of biological relations between individuals is fundamental to understanding past human societies. Caregiving, resource sharing and sexual behaviours are often mediated by biological kinship and yet the identification and interpretation of kin relationships in prehistoric human groups is difficult. In recent years, the advent of archaeogenetic techniques have offered a fresh approach, and when combined with more traditional osteological and interpretive archaeological methods, allows for improved interpretation of the burial practices, cultural behaviours, and societal stratification in ancient societies. Although archaeogenetic techniques are developing at pace, questions remain as to their accuracy, particularly when applied to the low coverage datasets that results from the sequencing of DNA derived from highly degraded ancient material.

**Results:**

The performance of six of the most commonly used kinship identifcation software methods was explored at a range of low and ultra low genome coverages. An asymmetrical response was observed across packages, with decreased genome coverage resulting in differences in both direction and degree of change of calculated kinship scores and thus pairwise relatedness estimates are dependant on both package used and genome coverage. Methods reliant upon genotype likelihoods methods (lcMLkin, NGSrelate and NGSremix) show a decreased level of prediction at coverage below 1x, although were consistent in the particular relationships identified at these coverages when compared to the pseudohaploid reliant methods tested (READ, the Kennett 2017 method and TKGWV2.0). The three pseudohaploid methods show predictive potential at coverages as low as 0.05x, although the accuracy of the relationships identified is questionable given the increase in the number of relationships identifIed at the low coverage (type I errors).

**Conclusion:**

Two pseudohaploid methods (READ and Kennett 2017) show relatively consistent inference of kin relationships at low coverage (0.5x), with READ only showing a significant performance drop off at ultralow coverages (< 0.2x). More generally, our results reveal asymmetrical kinship classifications in some software packages even at high coverages, highlighting the importance of applying multiple methods to authenticate kin relationships in ancient material, along with the continuing need to develop laboratory methods that maximise data output for downstream analyses.

**Supplementary Information:**

The online version contains supplementary material available at 10.1186/s12864-023-09198-4.

## Background

Over the past two decades, ancient DNA studies have made outstanding contributions to the understanding of historical human migrations that occurred on both a regional and continental scale [1–5]. However, as extraction and sequencing techniques improve and aDNA methods become both cheaper and more accessible, a renewed focus on individuals from a single site or region has developed. While this focus is often a component of larger studies [6], they are increasingly becoming the primary focus, for example with the identification of a kin-mediated burial in the Early Neolithic [7], social stratification of Irish Neolithic communities [8], social complexity in an Upper Palaeolithic community [9] and the most ancient, shared burial of monozygotic twins [10]. Much of the power of these studies derives from the ability to harness nuclear genome data to perform biological kinship calculations, which when combined with mitochondrial and Y-chromosome haplogroup information allows for the creation of complex pedigrees that further inform understanding of the social striation and cultural practices of historic cultures and societies.

The most common method of identifying biological pairwise relatedness in genetic studies is the calculation of the kinship coefficient ($\varvec{\varphi })$: the probability that a pair of homologous alleles at an autosomal locus are identical by descent (IBD) [11] and whose value is directly linked to the biological relationship between the two individuals (0.25: sibling/parent/offspring, 0.125: grandparent/aunt/uncle, etc.). When considering modern high quality human genomes, the abundance of phased autosomal data means the identification of these regions can allow for relatedness between individuals to be established, alongside ancestral signatures of admixture and inbreeding [12, 13]. Notably however, genome data sequenced from archaeological human remains is generally of lower coverage, with the mapping of ancient read data to the reference often resulting in an incomplete and unphased genome [14]. To address the problem of missing data, several ancient DNA specific software packages have been developed which look to combine statistical methods developed for ancient genome data processing with the mathematical framework for the identification of IBD regions, to calculate and identify kinship in low coverage data.

The majority of ancient DNA kinship estimation software packages calculate a pairwise $\varvec{\varphi }$ value but differ in terms of the statistical approach used to produce such a value. The most widespread approach treats the diploid human genome as haploid and performs pseudohaploid genotype calls across the genome [15]. This allows for genetic information to be obtained at a nucleotide site covered by only a single read, although this doesn’t consider the uncertainty of such a call [16]. The second, more recent development is the use of a genotype likelihood approach that estimates the likelihood of each allele (A, C, T or G) at a given nucleotide position, and which was developed to consider the uncertainty in a nucleotide call when sequence data is of low coverage [17]. Such methods are incorporated with kinship coefficient calculations to estimate a kinship score and allow for biological relationships to be identified in low coverage datasets.

Despite developments in ancient DNA recovery, datasets are still typically low in both data quantity and quality, resulting in low or even ultra-low genome coverage data (defined here as less than 2x mean genome coverage, and 0.5x mean genome coverage respectively). This problem is typically not a consideration during the development of aDNA specific kinship estimation software packages, with the focus instead on data of intermediate (2x and over) genome coverage.

Here, we compare the performance of six relatedness-inference software methods explicitly designed for ancient DNA data (NGSRemix [18], NGSRelate [19], lcMLkin [20], READ [21], TKGVW2.0 [22], and a method first described in Kennett et al. 2017 [23] and referred to from here on as the “Kennett method”), using three whole genome sequenced (WGS) ancient datasets showing different levels of relatedness in source publications [9, 24, 25] and a dataset of modern genomes containing related individuals obtained from the Gambian Genome Diversity Project [26]. Sequence data at various levels of down sampled coverages are used as input to detect the response of each package at a range of shared pairwise coverages (mean 2.1x to 0.02x coverage). Given the true relationships in the ancient datasets are unknown, rather than looking to identify specific relationships across coverage we look to identify the degree of kinship value change across coverages. We treat results from the highest coverage datasets act as a baseline to which all down sampled results are compared, with greater deviation at low coverages indicative of lower accuracy and/or consistency for the method. Sequence data from modern individuals of known pedigree are included to allow for false positive and negative rates to be calculated and support any findings from analysis of the ancient datasets.

## Results

**Fig. 1:**
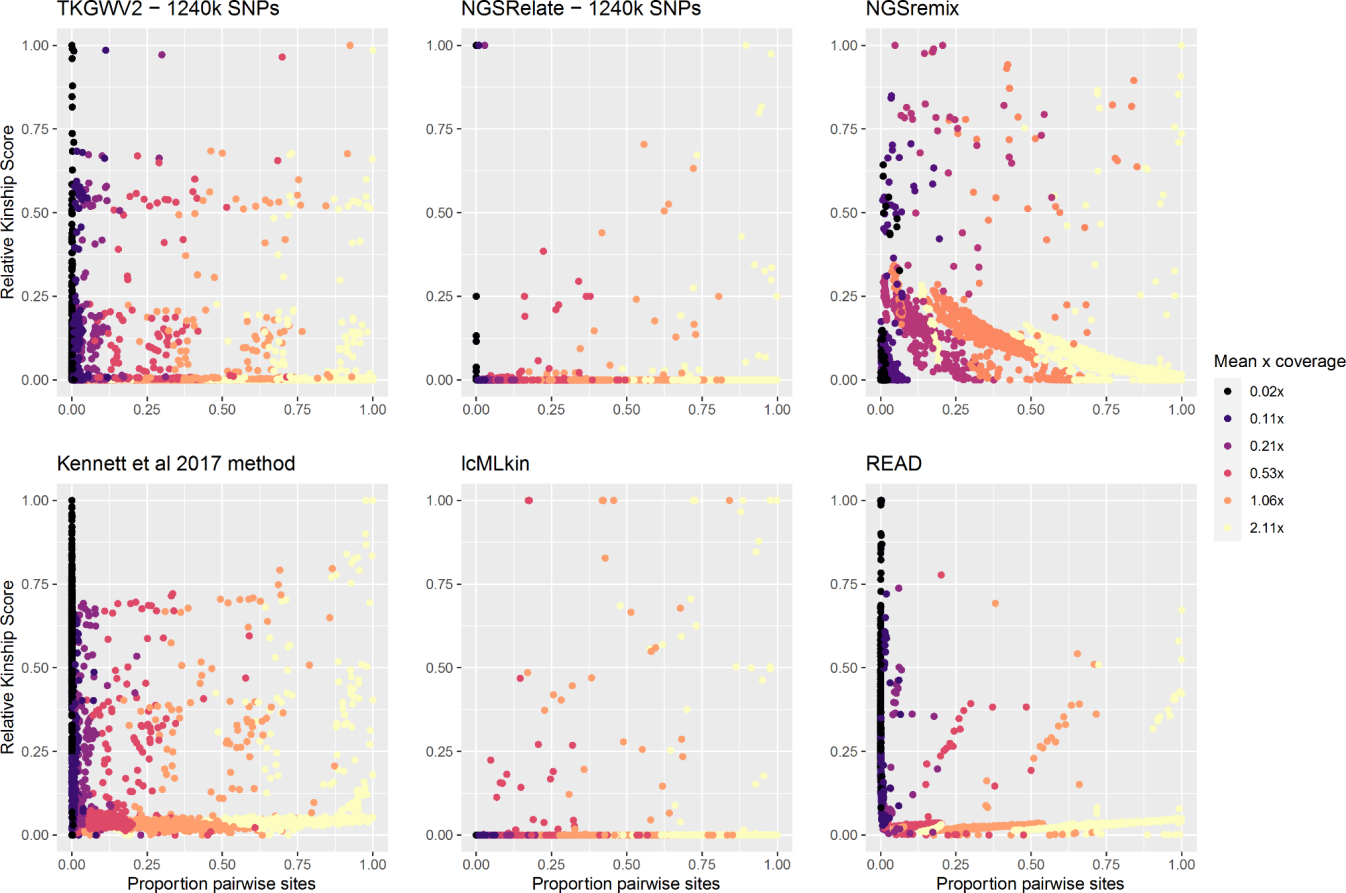
The effect of low coverage data on biological relationship calculations for the six relatedness methods compared in this study. Data from the three ancient datasets has been combined. Datapoint colours indicate the proportion of original reads sampled. Results of associated mantel tests can be found in Table S1.9. Details of the calculations to produce values corresponding to the proportion of maximum number of pairwise sites and the relative kinship score can be found in the supplemental information.

A total of 976 pairwise relationships were investigated, resulting in the calculation of 40 992 individual pairwise kin relationships across the six inference methods. Seven separate approaches (see material and methods) were used in this comparison, and all were successful in identifying biological relations within all datasets and across coverages (Fig. 1*).* All statistical tests reported in the main text have been performed on a combined dataset containing the results of analysis on the three ancient WGS datasets. Results for each dataset can be found in the supplemental information (Table S1.1-1.14), but mirror patterns seen in the combined WGS datasets. The mean genome coverage for all sequence data was calculated using coverage command in samtools v1.12 [27] (Table S1.15).

**Fig. 2:**
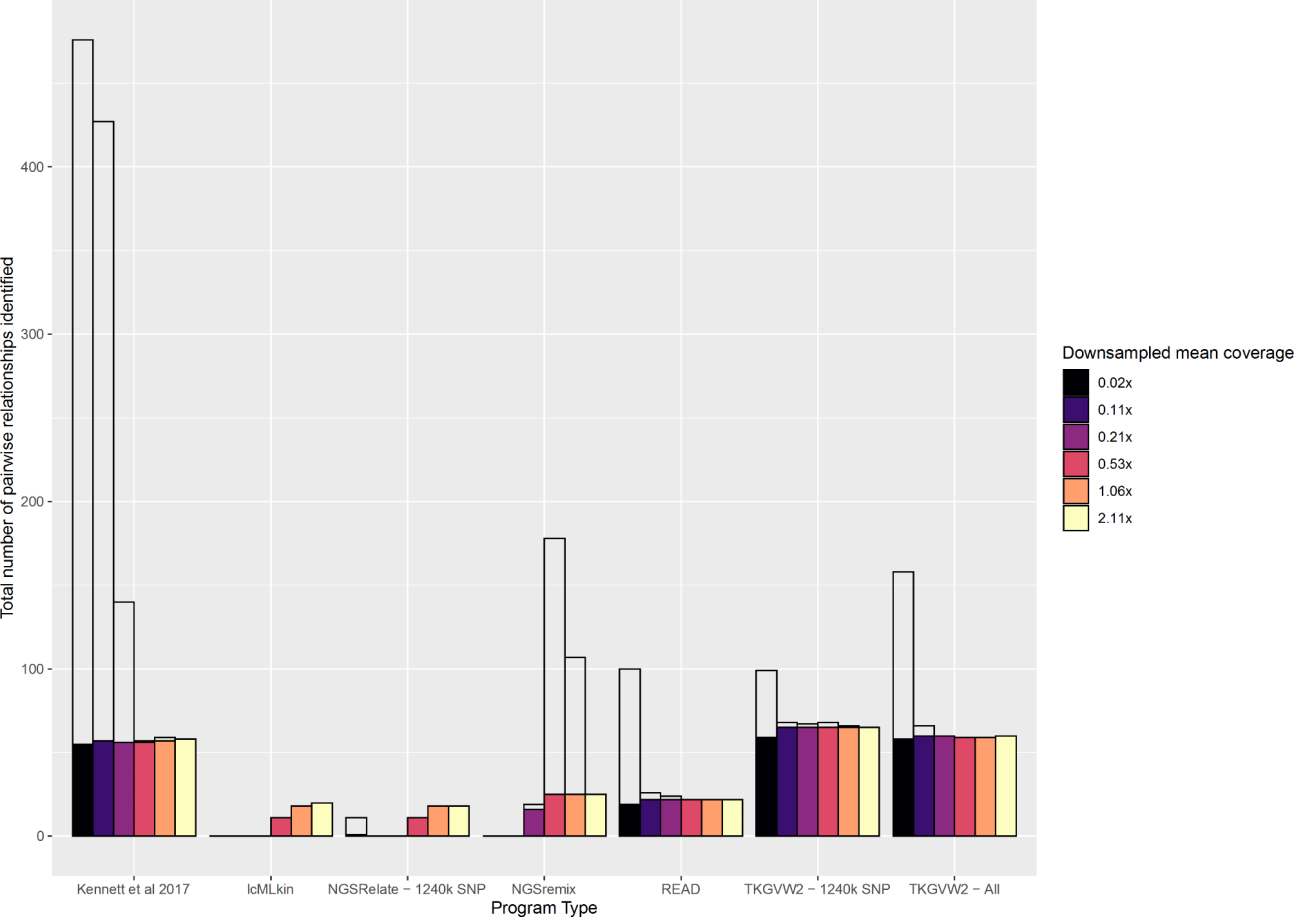
Variation in the number of pairwise relationships identified between packages and across coverages. Data collated from the three ancient datasets. Solid bars indicate the number of consistent relationships identified at maximum and reduced coverage (consistency/false negatives/type II error). Translucent bars indicate the number of relationships across coverages not identified at maximum coverage (Accuracy/false positives/type I errors).

Pairwise mantel correlation tests found genome coverage to significantly influence kinship inference (Fig. 1, Table S1.1-1.8) for the software packages lcMLkin (*Mantel R*: 0.13, *p*:0.01), NGSRemix (*Mantel R*: 0.04, *p*:0.01), NGSRelate (*Mantel R*: 0.08, *p*:0.01), the Kennett method (*Mantel R*: 0.07 *p* < 0.01) and TKGWV2.0 using both whole genome (*Mantel R*: 0.03, *p*:0.02) and SNP data (*Mantel R*: 0.03, *p*:0.01). No relationship between coverage and resulting kinship score was found for READ (Table S1.9; *Mantel R*: 0.03, p:0.39). Differing responses to low coverage data were seen both between and across pseudo haploid and genotype likelihoods methods (Fig. 1), with genotype likelihood methods showing a greater correlation (higher observed correlated R value) between genome coverage and kinship score than pseudo haploid methods (Table S1.9). The number of relationships identified across packages at the highest coverage was largely consistent, although TKGVW2.0 and the Kennett method predicted a greater degree of relatedness in two of the three ancient datasets (Fig. 2). Both identity and the number of related pairs differed from those found in the source publication on a number of occasions, the most significant of which being within the dataset containing 15 individuals from a 5,000-y-old mass grave [24] which reported 55 kin relationships on publication but ranged from 10 to 53 across the seven methods at maximum coverage in this study. The degree of kin relatedness reported in the source paper was most similar to the results provided by the Kennett method and the two TKGWV2.0 methods (Table S1.9).

**Fig. 3:**
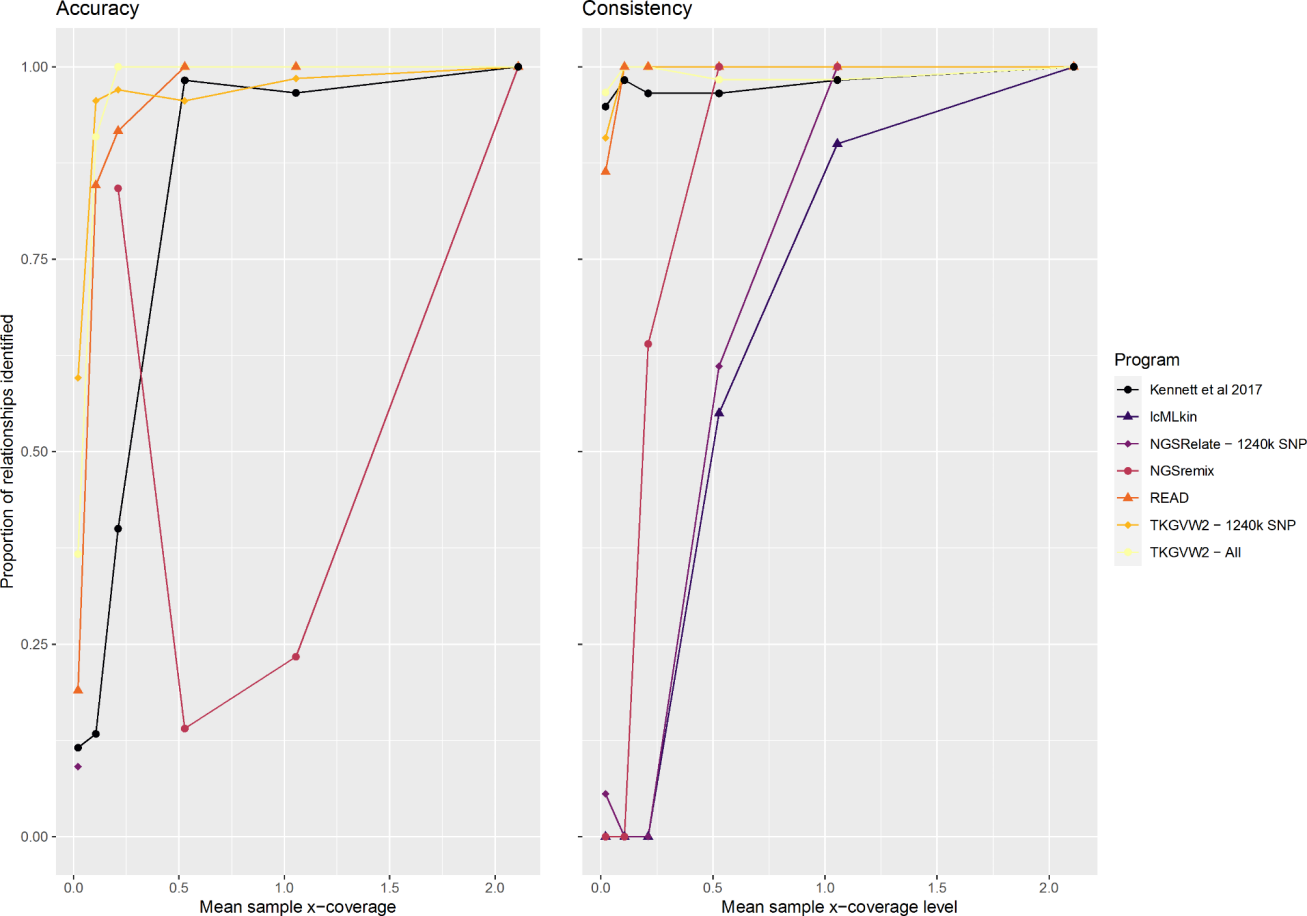
The accuracy and consistency (as defined in the methods) of inferred relatedness at all coverage levels for each calculation method. Combined data from the three WGS ancient datasets was used as input (Table S1.10-14)

The genotype likelihood dependant packages lcMLkin and NGSRelate showed a decline in the number of relationships inferred as coverage decreased (Fig. 2). All relationships that continued to be inferred at reduced coverage were among those inferred at the highest coverage (Fig. 3). NGSRemix responded to decreasing genomic coverage by overpredicting the number of kin relationships identified at intermediate genome coverages, before dropping sharply at ultra-low coverages (Fig. 2). At maximum coverage, it performs similarly to other genotype likelihood methods, with consistency seen in the specific pairwise relationships identified between these packages (Fig. 2, Table S1.10-13). The R0 (*Adj. R*^*2*^: 0.4436; *p*:<0.001), R1 (*Adj. R*^*2*^:0.467; *p*:<0.001) and KING (*Adj. R*^*2*^: 0.0143; *p*:<0.001) summary statistics calculated through NGSRelate (Table S1.3) were found to be significantly affected by genome coverage, with variance increasing as pairwise coverage decrease (*Fig. S1)*.

The number of biological relationships inferred by TKGVW2.0 was found to be consistent across all but the lowest coverage (Fig. 1), although there was inconsistency in terms of the specific individual pairs being identified as related across these coverages (Table S1.9-1.13, Fig. 3*)*. Despite the number of relationships remaining consistent across coverages, the number of specific relationships consistent with those inferred at the highest coverage level decreased as genome coverage decreased (Fig. 3). This may explain why no variation in kinship values produced by TKGVW2.0 are seen across genomic coverages (Fig. 1). Further, the number of kin relations identified for two of the three ancient datasets (Fig. 2) were higher than that seen in the four other approaches at the highest coverage.

The ability of READ to infer pairwise relationships showed an intermediate response to decreasing coverage (Figs. 1 and 2), and no correlation was identified between kinship score and genome coverage (Table S1.8). Only at ultra-low coverage does READ begin to function sub-optimally, with the consistency of relationships (when compared to the highest coverage) identified (type I error) rising ( Figs. S2 and S3). Results from the Kennett method largely mirror those of READ, with genome coverage having little effect on performance except at the lowest two coverage class (Figs. 1 and 2; Figs. S2 and S3). The results of the Kennett method diverge from that of READ when applied to a dataset that consisted of five individuals from the Upper Palaeolithic that were previously reported as unrelated [9]. The Kennett method identifies 7 relationships at the highest coverage, a result only mirrored by the output of TKGWV2 (which is deemed inaccurate due to non-concordant allele frequencies). No other method identifies relatedness within this dataset.

Modern data was incorporated to corroborate results of the ancient datasets for individuals of known relationships and identify the impact of damage on kin estimation. False negative and false positive rates are calculated, given the knowledge of true relationships (which is not known in the ancient datasets). The responses of each package to decreasing genome coverage largely mirrors that seen in the ancient datasets (Table S1.14; Figs. S2 and S3), with genotype likelihood methods lcMLkin and NGSRelate showing an increase in false negatives and thus underprediction at low coverage, and pseudo haploid methods (READ, TKGVW2.0 and the Kennett method) showing an overprediction of kin relationships at the lowest of coverages (false positives). However, in contrast with analysis of the ancient dataset, no disparity was identified in the number of relationships inferred at the highest coverage across packages (Fig. S2) and no overprediction was seen when NGSremix inferred relationships at intermediate coverages (Figs. S2 and S3).

## Discussion

Asymmetrical responses to decreasing coverage can be seen across the six software methods. When considering the modern data, lower coverage data results in an increased proportion of false negatives (type II error) in the programs utilising genotype likelihood methods (NGSRelate, NGSremix and lcMLkin), whilst programs that use pseudo haploid calls for kinship classification (TKGVW2.0, READ and the Kennett method) have an increased number of false positives (type I error) (Figs. S2 and S3). This response to low genome coverage is mirrored in the ancient datasets, with genotype likelihood methods showing a drop in the accuracy and number of inferred relationships, and pseudo haploid methods showing a drop in the consistency (and overprediction at the lowest of coverages; Figs. 2 and 3). In general, pseudo haploid methods are shown to maintain performance (especially READ) at all but the lowest of coverages. The symmetry seen in package response across modern and ancient data indicates that the impact of DNA damage is negligible when compared to the impact of genome coverage.

We might expect that all genotype likelihood methods would respond in a similar manner to decreasing genome coverage, given that they are largely based upon the same statistical framework, and this is confirmed with results here, where prediction potential is seen to drop as genome coverage reduced. This is likely a product of the increased uncertainty associated with genotype likelihood calls. The likelihood of a nucleotide being of a particular state (A, T, C or G) is calculated at each nucleotide site in the genome and is dependent on the number of reads and thus depth of coverage at each site. The lower the coverage at a particular site, the greater uncertainty there is as to the true allelic state at this nucleotide position. The increased allelic uncertainty at each site is compounded when considering pairwise relationships using low coverage data, with the probability of two individuals sharing a specific allelic position declining as coverage decreases, and thus fewer shared sites for use in calculation.

The kinship coefficients produced by NGSRelate and lcMLkin appear accurate when mean pairwise genome coverage is over 2x, and it is thus proposed that such packages are reserved for higher coverage ancient data, although the lack of false positives even at the lowest coverage would suggest that even for low coverage data these methods could be considered useful when the correct identification of a specific kin relationship is of particular importance. The R0, R1 and KING values calculated within NGSrelate show increased variance as coverage decreased (Fig. S1), although unlike the calculated $\varvec{\varphi }$ from this package, an increase in variance of these values would lead to incorrect kinship classification making this method less robust to missing data and more likely to result in type II error.

NGSRemix is anomalous in its response to decreasing coverage, with the overestimation of kin relations seen at intermediate genome coverage. This is a likely to be associated with its reliance on the ADMIXTURE package which requires data of sufficient quality to allow for the accurate determination of the number of admixed elements within an individual’s genome [28], with the accurate identification of the number of ancestral proportions a requirement for accurate NGSremix kin calculations. Whilst identifying and incorporating admixture information into a biological kinship calculation is undoubtedly useful and applicable when high coveage data is avaialble, its dependance on ADMIXTURE is a severe limitation and makes it unsuitable for ultra-low coverage data.

The three methods (READ, TKGVW2.0 and the Kennett method) that use pseudohaploid calls appear to be more robust in terms of the variation seen in relative kinship scores as coverage decreased. However, although the number of kin relationships identified is largely consistent across coverage levels, this was largely due to an increase in false positives coupled with a proportional decrease in false negatives. At the lowest coverage level (0.02x mean sample coverage) all programs overpredict the number of relationships identified, leading to a drop in consistency and accuracy of kin relationships identified, and an increase in false positives (type I error).

Of the three pseudohaploid programs tested, our study identified READ as the most reliable, identifying a similar number of relationships as the likelihood methods at high coverage, and showing reasonable consistency in the specificity of relationships identified as coverages decreases. The program employs a normalisation step after pairwise shared alleles have been identified that was developed to overcome issues associated with missing/limited data [21]. This normalisation calculation is solely dependent on the input data and looks to identify the expected number of shared alleles between unrelated individuals. Therefore, even at low pairwise genetic coverage, it identifies the proportional differences in the shared number of alleles seen within the group rather than directly attributing the number of shared alleles to a specific kinship classification. Any pairwise kinship score that differs significantly from the expected value of a non-related pair is thus identified as related. It is this within-group comparative method that is the major novelty of the program and may explains how it maintains relative consistency across the range of coverages.

The Kennett method performs similarly to READ in its accuracy and consistency across the four highest coverage levels, but overpredicts relationships at the lowest of coverage levels (0.02x and 0.11x mean coverage). Notably, the software performs to varying degrees of success across datasets, with a significant relationship between genome coverage and output kinship score in two of the three ancient datasets (Table S1.10) that is absent when analysing the two other datasets. Like READ, it relies upon a normalisation step solely dependent on the specific pairwise mismatch rate calculated from input data, although the calculation method of this value differs. The highest mismatch rate is predicted to be that of two unrelated individuals, with any significant deviation from this rate showing evidence for more shared pairwise sites and thus a greater degree of relatedness. Use of this maximum mismatch rate in kinship calculation allows for the calculation of the relatedness coefficient, with the degree of mismatch rate difference proportional to biological relatedness. In principle this method is sound, however in practice it struggles when no individuals within a test dataset are related. An example of this can be seen with the Upper Palaeolithic dataset [9], which was predicted to contain no related individuals by all calculation methods except the Kennett method, which predicted biological relationships across all coverage levels. Such overprediction can be easily avoided however with the initial examination of pairwise mismatch scores. If no significant difference is seen across mismatch rates, it can be assumed that no kin relationships exist with the dataset (or a far less likely scenario that all individuals are related to the same degree). Although both READ and Kennett methods use a normalisation step, READ is more robust across genome coverages given the relationship seen between genome coverage and kinship score for two of the four datasets. On investigation, this is likely due to the overprediction by Kennett at the lowest coverages (Table S1.13) and may stem from a normalisation step that considers only the highest pairwise mismatch rate value, which would become less precise as data is removed.

Perhaps the most notable shortcoming of both READ and Kennett methods is the requisite for a dataset of sufficient sample size to allow for both a reliable maximum pairwise and normalisation value of the population to be calculated. There is potential to overcome this by allowing for the manual input of normalisation values, but this requires knowledge of a reliable pairwise mismatch rates of the population being studied, which is difficult to predict when considering ancient human datasets.

TKGWV.2 is based upon pseudohaploid diploid genotype calls, but rather than solely using these to infer related individuals, it uses a given set of expected allele frequencies at shared nucleotide positions. This provides a reference of allele frequencies that are expected to be found within the population, and thus allows for the probability of two individuals sharing the same allelic state to be determined and fed into the kinship calculation. These allele frequencies add significant power to the analysis, and thus far fewer shared nucleotide positions are required for a kinship classification to be ascertained. In the dataset containing Viking individuals (where the allele frequencies provided were expected to be comparable with those found in the population under study) TKGWV.2 performs strongly, with consistent predictions down to 1% of the original BAM file (0.02x mean coverage). However, when the allele frequencies used are not representative of the ancient population (as seen in the UP dataset), there is significant over-representation of the kin relationships. This is to be expected, and highlights the importance of using a set of allele frequencies that are representative of the group being studied [22]. When considering ancient populations, accurate pre-Neolithic allele frequency information is lacking due to the sparseness of high coverage genomes needed for accurate allele frequencies to be calculated. We therefore suggest that this program be reserved for more contemporary populations for which accurate allele frequencies can be calculated.

An unforeseen outcome of this study was the reassessment of studies that have previously employed a single software package to calculate kinship. Of the three ancient datasets used, results from the Koszyce dataset are particularly striking, with fewer kin relationships identified when using 3 out of the 6 kinship methods than in the initial publication [24] (40 1^st^ or 2^nd^ order relationships in the source publication vs. 12 to 58 when using alternative packages). This serves to highlight how genetic relatedness predictions at low coverage can be significantly affected by the method of calculation and highlights the importance of using multiple independent methods to ameliorate any software specific issues, and best allow for the inference of true relationships in archaeological studies.

## Conclusion

The ability to determine a biological relationship in ancient populations is a crucial tool for establishing cultural histories. Here we have highlighted that such identification is dependent on both the statistical method used and the pairwise genome coverage of the data. Genotype likelihood methods are robust to 2x mean coverage, and rarely succumb to false positives as coverage decreases. Pseudohaploid methods can identify relationships at coverages as low as 0.02x mean genome coverage, but at these ultra-low coverages false positives are increasingly abundant. READ is the most suitable method at low to ultra-low coverages (and to a lesser extent the Kennett method), but no single package is identified as best across all coverages. To best overcome the disparity seen at low coverage, we suggest the use of multiple kinship inference packages that use distinct calculation methods to best corroborate any kin relationships seen. What is clear from this study is that no method can sufficiently overcome a lack of and/or poor-quality sequence data, highlighting that the consistent developments of novel pre- and post-sequencing aDNA methodologies (robust capture methods, genome imputation etc.) must be sustained over the coming years to maximise the quality and quantity of ancient sequence data.

## Methods

Four datasets of whole genome sequence (WGS) read data were used in this study, and run through identical computational pipelines developed expressly for each software package/method.

### Ancient genomic datasets

Pre-aligned ancient read data from three independent sources were downloaded from the ENA database. All data had passed through authentication procedures that confirmed the DNA as being genuinely ancient [9, 24, 25, 29], and laboratory methods followed standard ancient DNA extraction, library building and sequencing protocols (outlined briefly in the supplemental material). All sequence data had been aligned to the GRCh37 reference genome and downloaded in the BAM format.

The three datasets containing genome wide sequence (WGS) data:

1. 15 individuals from a single Late Neolithic mass grave (Koszyce) in Poland (~ 5kya) showing a varying degree of biological relatedness between individuals [24];
2. 5 unrelated individuals from Sunghir, an Upper Palaeolithic cave site dated to around 34kya located in Western Russia [9];
3. 28 individuals from a single Viking burial on the island of Saaremaa in Estonia (~ 1.3kya) who showed evidence for biological relatedness amongst individuals [25].

Details of the archaeological context for these three datasets, and the methods used to identify kin relationships in the source publication can be found in the supplemental material.

### Modern genomic data

A modern genomic dataset was also included in this study to permit the analysis of individuals known to be related. Unaligned paired end sequence data of 15 Fula individuals from the Gambian Genome Diversity Project (GGVP) GCh37 were downloaded from the 1000Genomes Project Phase 3 database [26]. The fifteen individuals belonged to five discrete family units (father, mother, offspring) who were otherwise unrelated to all others.

Downloaded fastq sequence files were processed using ancient DNA specific software packages to provide a degree of conformity in the handling of both the modern and ancient datasets. Paired end reads were collapsed using AdapterRemovalv2 [30] under default settings, and aligned to human reference genome GCh37 using the aln function of bwa v0.7.17 [31]. Samtools v1.12 [27] was used to sort, remove duplicates, and quality filter (Q ≥ 30) bwa output files, and resulting in the creation of 15 BAM files containing modern sequence data curated to mirror the ancient datasets. Both transition and transversion sites were used for analysis.

### Analytical pipeline

Both ancient and modern datasets were treated independently and followed a standardised computational and analytical pipeline. Each BAM file was down sampled to 1%, 5%, 10%, 25% and 50% of all reads present in the original (100%) BAM file, with the coverage of all resulting BAMs determined using samtools v1.12 (mean genome coverage when combining all ancient WGS data: 100% = 2.12x, 50% = 1.06x, 25% = 0.53x, 10%= 0.21x, 5% = 0.11x and 1% = 0.02x). Exact coverage details for all sequence files can be found in the supplemental information (Table S1.15).

These down sampled BAM files were indexed using samtools v1.12, and input into computational pipelines that had been created for each of the six relatedness methods being compared: NGSRemix [18], NGSRelate [19], lcMLkin [20], READ [21], TKGVW2.0 [22] and the Kennett method [23]. These six methods can be grouped into two categories: those using pseudohaploid genotype calling methods for biological kin identification, and those using genotype likelihoods. All analysis (unless otherwise stated) was restricted to 1240k nucleotide sites previously identified as polymorphic in ancient populations [32, 33] to best overcome any issues with missing data and standardise all calculations across datasets and coverages.

### Pseudohaploid genotype methods

#### READ

The READ package leverages genomic regions that are identical by descent (IBD) to calculate biological relationships between individuals. Here, with pseudohaploid data as input, each genome is divided into 1 million base pair regions and the proportion of non-matching alleles within each region is calculated (P0). This is repeated across the genome and for all possible pairwise comparisons. A normalisation step is then undertaken using the expected P0 values of a pair of non-related individuals within the group being analysed, resulting in a final relatedness prediction that is independent of within group genetic diversity, SNP ascertainment biases and marker densities variation [21]. The package offers three options for such normalisation step, with this study using the default mean P0 method (the mean P0 across all individuals) as the majority of individuals within each dataset were deemed to be unrelated in the source publication. After normalisation has occurred, a kinship classification (either the first- or second- degree) is made using the normalised P0 score for each pairwise comparison.

A genotype calling method implemented using the pileupCaller from the software package sequenceTools [34] was used to provide pseudo haploid genotype call data. The allelic state of a randomly selected read was sampled at each nucleotide site, resulting in a haploid genome representative of the read data input. This random approach was used to mitigate the potential reference bias intrinsic to a consensus calling method. A pipeline combining samtools mpileup, and pileupCaller was used to carryout genotype calling, with genotypes being called at the 1240k nucleotide site list. This was to standardise the number and location of SNPs being used between datasets and coverages to reduce the impact of linkage disequilibrium, and thus allow for direct comparison between datasets. EIGENSOFT function convertf v8.0.0 [35, 36] and PLINK version 1.90b6.10 [37] was used to convert pileupCaller format files to the required format for READ (recode tped/tfam). READ was run using default settings and the standard mean normalisation step.

#### TKGWV2.0

The TKGWV2.0 package requires the user to provide a set of allele frequencies that correspond with those expected in the ancient population being analysed [22]. First, pseudohaploid genotype calls are made for a user determined set of nucleotide loci at polymorphic nucleotide sites (where a read is selected randomly at each site to avoid reference bias) before the identification of shared sites between all individual pair combinations. The expected allele frequencies at each of these sites is then leveraged from the set of allele frequencies provided by the user, before a pairwise relatedness estimation is performed (supplemental information). Such estimation is reliant on IBD regions and produces halved relatedness coefficients (due to the haploid nature of the data) to allow for biological relatedness to be determined. This **HRC** (halved relatedness coefficient) value is analogous to the kinship coefficient $\varvec{\varphi }$ (**HRC**= $\varvec{\varphi }$ x 2).

TKGWV2.0 was installed through GitHub and run with default settings using allele frequencies generated from modern European CEU individuals sequenced for Phase 3 of the 1000 Genomes Project [26] for the three ancient datasets, and a set of allele frequencies calculated from 100 Fula individuals from the Gambian Genome Diversity Project (GGVP). Two iterations of the analysis were run; the first used 22 million SNPs identified as polymorphic in modern populations (Auton et al., 2015), the second using the set of 1240k SNPs. Results for both methods were similar (Table S1.3-4), and thus only the 1240k SNP results were incorporated into the main text and figures.

### The “Kennett method” [23]

The Kennett method was first applied in 2017 [23] and has been widely used in subsequent publications looking to identify biological kinship in ancient populations (7,35,36, etc.). It leverages pseudohaploid genotype calls and is targeted at data that has been generated using SNP methods. Calculation is a two step process. The first requires the calculation of pairwise mismatch rates across all samples, and subsequent examining of these values to identify any significant difference between values across the dataset. If no significant difference in pairwise mismatch rate is observed, then an assumption is made that no individuals are related, with the same degree of allele sharing across all individuals. If pairwise mismatch rates are found to differ significantly, then allele sharing is non uniform between individuals and thus a degree of relatedness is predicted within the group. Such data is thus carried forwards to a second step, which uses the maximum mismatch rate as a normalisation value (in a similar manner as READ) for relatedness coefficient prediction (described in the supplementary text).

There is currently no publicly available software that implements the Kennett method but given its prevalence in the literature it was incorporated into this study. GATK was used to call genotypes, PLINK used to calculate mismatch rates and base R used to manipulate data and calculate the kinship coefficient.

### Genotype likelihood methods

#### lcMLkin

lcMLkin uses genotype likelihoods to calculate the k_0_, k_1_ and k_2_ summary statistics, that when combined allow for the calculation of the kinship coefficient $\varvec{\varphi }$.

A genotype likelihood calling method was implemented in GATK version 4.1.9.0 [38]. The HaplotypeCaller, CombineGVCFs and GenotypeGVCFs software tools within GATK were used to calculate genotype likelihoods at the 1240k nucleotide site list. This was to standardise the number and location of SNPs being used between datasets and coverages to reduce the impact of linkage disequilibrium, and thus allow for direct comparison between datasets. GATK provided variant call files (VCFs) that contained genotype likelihoods information suitable for use in the three genotype likelihood methods: NGSrelate, lcMLkin and NGSremix.

lcMLkin was run with genotype likelihoods calculated using the GATK pipeline as described, with the output VCF files being input into lcMLkin using the phred-scaled likelihoods option (-g phred). The package provides a subsidiary script (BAM2VCF.py) to call variants from a set of BAM files, but after preliminary testing this method was abandoned as the computational requirements were too great for analysis required for this study. This left GATK as the sole genotype likelihood estimation package used in this study.

#### NGSRelate

NGSRelate requires genotype likelihood data as input, and is reliant on the computation of nine Jacquard coefficients [39] that can be used for the calculation of a range of summary statistics that include the determination of $\varvec{\varphi }$. Each of the nine Jacquard coefficients are dependent on the specific assortment of alleles that can be produced in the offspring of two diploid individuals, of which there are 15 in total. If the paternal and maternal origin of each allele are discounted as uninformative, nine of particular significance in biological heredity calculations remain. Alongside the $\varvec{\varphi }$ calculation, NGSRelate uses an additional method to infer the relatedness of individuals through alleles that are identical by state (IBS) and 2-dimension site frequency spectrum (2D SFS). This method uses three summary statistics, the R0, R1 and KING scores as reported here: Relatedness predictions are carried out by exploiting the relationships between R0, R1 and KING values: R1/KING and R0/KING [40], with the distribution of these ratios allowing for biological relatedness between individuals to be predicted through comparison to previously determined threshold values, which correspond to specific biological relations.

NGSRelate is a subsidiary package of ANGSD [16] and requires genotype likelihoods and allele frequencies as input. These genotype likelihoods and allele frequency calculations were performed using ANGSD version 0.929 at 1240k SNP sites. Outputs were parsed to NGSRelate and kinship coefficients calculated.

#### NGSRemix

NGSRemix is a software package designed to allow for the calculation of kinship coefficients in admixed populations [18] using the program ADMIXTURE, which determines population dependant allele frequencies and individual ancestry proportions [28, 41]. For standard allele frequency kinship methods, allele frequencies are calculated across a genome and thus do not consider the potential for asymmetrical allele frequencies if individuals were recently admixed and thus contained ancestral components from two genetically distinct populations with nonconforming allele frequencies. This method seeks to determine the number of ancestral populations that contributed to an individual’s genome, and the precise allele frequencies of each ancestral component.

NGSRemix requires genotype likelihoods as input, which were calculated using the GATK pipeline as described previously. PLINK version 1.90b6.10 [37] was used to manipulate files and produce the required file format for input into ADMIXTURE version 1.3.5 [28], which was run using default settings. ADMIXTURE is essential to this method, as it determines the number of ancestral source populations, the specific ancestry components, and the population specific allele frequencies for each individual. Although all datasets used were known to not contain any admixture individuals, a range of *K* values (number of ancestral source population) between 1 and 10 were used as input into ADMIXTURE to confirm this. Allele frequencies calculated from the *K* value (the lowest CV error) deemed most suitable at high coverage datasets was used as input into the NGSRemix program. NGSRemix was run using default settings.

Whilst six software packages were compared, we also included additional approaches within the packages where appropriate. This led to a total of seven approaches being used to calculate biological kinship: NGSRemix using default parameters, NGSRelate at informative 1240k SNP sites, lcMLkin at informative 1240k SNP sites using default parameters, TKGWV2 using a SNP panel containing 22mill polymorphic sites and at informative 1240k SNP sites, READ using default parameters and the Kennett method at informative 1240k SNPs. Datasets were treated separately during computational analysis before data from the three ancient datasets were combined for the final statistical analysis and visualisation. To allow for direct comparison between software packages, a threshold for biological relatedness was set at the 2nd degree biological relationship, and a binary kinship distinction used to allow for comparison between packages: two individuals were either related (1st or 2nd degree) or unrelated.

### Statistical analysis

Statistical analysis was carried out to determine the effect of pairwise site coverage on the determination of pairwise relatedness. A relative kinship score was calculated (see supplemental information) for every pairwise comparison, and pairwise mantel tests performed in R version 4.1.1 [42] for every software package and input data iteration, with relative kinship score modelled against pairwise site coverage. The three ancient WGS datasets were combined for the final analysis and visualisation.

To identify how consistent each package was across coverage levels, measures to determine the accuracy and consistency of each packages were calculated across all datasets (Fig. 3, Table S1.10-14):

**Accuracy** = # relationships identified at [x] coverage level that are consistent with those identified at maximum coverage level / total # of relationships identified at [x] coverage level.

**Consistency** = # relationships identified at [x] coverage level that are consistent with those identified at maximum coverage level / total # of relationships identified at maximum coverage level.

**False positives** were defined as the number of specific relationships at [x] coverage not identified at maximum coverage in the modern dataset. **False negatives **were defined as the number of specific relationships at maximum coverage not identified at [x] coverage.

All analyses were carried out on the high-performance cluster at the Natural History Museum (124 CPU’s, 2.256 TB RAM) with data visualisation performed in R version 4.1.1 [42]. All associated code for computational analysis is documented in the supplementary information.

## Electronic supplementary material

Below is the link to the electronic supplementary material.

**Additional file 1**: Identifying biological kinship in ancient datasets of ultra-low coverage: a comparison of aDNA specific software packages. **Figure S1**. The effect of low coverage data on biological relationship calculations using the R0, R1 and KING ratio method (3). **Figure S2**. Variation in the number of pairwise relationships identified between packages and across coverages. **Figure S3**. The consistency and accuracy (as defined in the methods) at all coverage levels for each kinship calculation method.

**Additional file 2**: **Table S1.1**. Results from all datasets using program lcMLkin and autosomal sites from the 1240k SNP panel. **Table S1.2**. Results from all datasets using program NGS relate and all shared autosomal sites. **Table S1.3**. Results from all datasets using program NGSrelate and autosomal sites on from the 1240k SNP panel. **Table S1.4**. Results from all datasets using program NGSremix and autosomal sites on the 1240k SNP panel. **Table S1.5**. Results from all datasets using program TKGWV2 and site from the 1240k SNP panel. **Table S1.6**. Results from all datasets using program TKGWV2 and autosomal sites from the panel of 22M SNP sites as described in the text. **Table S1.7**. Results from all datasets using program READ and autosomal sites from the 1240k SNP panel. **Table S1.8**. Results from all datasets using the Kennett method and autosomal sites from the 1240k SNP panel. **Table S1.9**. Mantel tests for all packages and datasets. **Table S1.10**. Accuracy, consistency and false positive calculations for the Koszyce/Schroeder dataset. **Table S1.11**. Accuracy, consistency and false positive calculations for the UP/Sikora dataset. **Table S1.12**. Accuracy, consistency and false positive calculations for the Viking/Willeslev dataset. **Table S1.13**. Accuracy, consistency and false positive calculations for the three WGS datasets. **Table S1.14**. Accuracy, consistency and false positive calculations for the modern/Fula dataset. **Table S1.15**. Coverages for all downsampled datafiles as calculated using samtools.

## Data Availability

All genetic data used in this study were accessed from open access depositories.  Details of computational pipelines and results of all analyses performed can be found in the supplementary materials. Ancient data can be accessed from the European nucleotide archive under accession numbers: PRJEB22592, PRJEB2845 and PRJEB37976. The modern Fula dataset can be accessed from the 1000 Genomes, Gambian Genome Variation project (GRCh37) project: https://www.internationalgenome.org/data-portal/population/GWF.
