# Supplementary material for: Inferring biological kinship in ancient datasets: comparing the response of ancient DNA-specific software packages to low coverage data
Source: BMC Genomics. 2023 Mar 14;24:111. doi: 10.1186/s12864-023-09198-4 (PMC10015695; doi:10.1186/s12864-023-09198-4)
Supplement: Supplementary file 1 — Additional file 1: Identifying biological kinship in ancient datasets of ultra-low coverage: a comparison of aDNA specific software packages. Figure S1. The effect of low coverage data on biological relationship calculations using the R0, R1 and KING ratio method (3). Figure S2. Variation in the number of pairwise relationships identified between packages and across coverages. Figure S3. The consistency and accuracy (as defined in the methods) at all coverage levels for each kinship calculation method. [file 12864_2023_9198_MOESM1_ESM.docx]

**Identifying biological kinship in ancient datasets of ultra-low coverage: a comparison of aDNA specific software packages - Supplemental Text**

**Datasets**

**Ancient datasets**

1. *Unraveling ancestry, kinship, and violence in a Late Neolithic mass grave* (1)

﻿A study focusing on a Late Neolithic mass grave located in southern Poland and containing individuals of the Globular Amphora culture. Sequence data obtained from the remains of 15 men, women, and children who all show blows to the head indicative of violent cause of death. Genomes were sequenced to between 1.1x and 3.9x fold coverage, with kinship analysis determining that most individuals belonged to an extended family, and that burial location was dependant on these kin relationships. The grave site is date to ~5kBP. It has been postulated that this grave consists of individuals killed during conflict between Globular Amphora and Corded Ware groups. Corded Ware groups expanded during this period, into regions neighbouring their prior home range that include this region of southern Poland (1).

Biological relatedness in the study was calculated using a method based on pairwise sharing of allele identical by state (IBS) and was performed using the ANGSD and NGSrelate software packages. Kin relations were determined using the KING (2), R0 and R1 ratios (3). Three family groups are identified containing 7, 3 and 3 related individuals respectively. Two individuals within the dataset share no biological kin relation below the third degree but do share a wider genetic affinity with the Globular Amphora population.

1. *Ancient genomes show social and reproductive behaviour of early Upper Palaeolithic foragers* (4)

A study of six human genomes sequenced from material excavated from the Upper Palaeolithic grave site of Sunghir located in Western Russia. Skeletal material was dated to ~34kya and included anatomically modern humans. Six specimens were destructively sampled for DNA extraction, with five of these returning enough endogenous DNA for subsequent bioinformatic methods (SI, SII, SIII, SIV and SVI). The sample SV did not provide enough genomic data for analyses, and hence was not included in this study. Genome coverages of the five individuals ranged from 1x to 10.75x. No biological relationships were found between these five individuals (1^st^, 2^nd,^ or 3^rd^ degree), signalling that population size was greater than initially believed, and that social organisation must have been suitably complex to avoid close kin relations within a residential group, and thus mating networks were regional in scale.

Kinship coefficient calculations were carried out using NGSrelate (5), alongside the calculation of KING (2), R0 and R1 ratios (3).

1. *Population genomics of the Viking world* (6)

A vast study containing 442 human genomes sequenced from numerous archaeological sites associated with the Viking period (AD 750-1050) across Scandinavia and Northern Europe. Data used within this kinship comparison came from a subset of these 442 genomes: a single Viking boat burial on the island of Saaremaa in Estonia and dated to around 1.3k±100 BP. 28 individuals were sequenced to sufficient quality to allow for kinship analysis in the source publication, with coverages of the 28 genomes ranging from 0.16x to 1.98x average depth of coverage. This ultra-low coverage genome (0.16x) was retained in the analysis to maintain dataset completeness. Two sets of brothers (four relatives total) were identified from this single burial in the source publication, with NGSrelate and ANGSD being used to calculate these relationships following standard settings.

The read data for the three datasets above was obtained using whole genome sequencing methods and standard ancient DNA extraction, library building, and sequencing methods as detailed in the source publication.

**Modern Dataset**

The modern data used in this study was downloaded from the Gambian Genotype Diversity Project, a subproject of the 1000 Genomes Project Phase 3 (7). The 15 individuals selected belonged to the Fula population of Western Gambia, a dataset within the Gambian Genotype Diversity Project (https://www.internationalgenome.org/data-portal/population/GWF). These 15 individuals comprised of 5 genetically distinct families, each consisting of a father, mother, and offspring trio. Biological relationships were ascertained in the original project using both genetic and social data.

Accession numbers for both modern and ancient data can be found in the accompanying supplementary excel documentation (S1.1-14).

**Computational Analysis**

**Alignment pipeline**

All ancient data used within the study was downloaded from the European Nucleotide Archive (ENA), had been previously aligned to human reference genome GCh37 (hg18) and following alignment protocols designed for ancient DNA data (1,4,8). All data was in the BAM format.

The modern data was downloaded as raw fastq files containing sequence data, and aligned in a similar manner to that used for the ancient data to mitigate any potential treatment biases. Alignment was carried using an aDNA specific bioinformatic pipeline at the Natural History Museum, London by W.M. In summary, the paired end fastq files were collapsed and combined using AdapterRemoval version 2.0 (9), before being aligned to the GCh37 using the aln function of bwa version 0.7.17 (10). Samtools version 1.10 (11) was used to sort BAM files, remove duplicates, and filter for quality (Q≥30) resulting in the production of 15 BAM files containing modern sequence data.

**Software settings and associated code**

Human reference genome GRCh37 was used as the default reference genome, and the 1240k SNP panel as the default list of nucleotide sites (12,13).

GATK

The HaplotypeCaller, CombineGVCFs and GenotypeGVCFs software tools within GATK were used for genotype likelihood calculations (for lcMLkin and NGSremix):

gatk --java-options "-Xmx4g" HaplotypeCaller -R [Homo_sapiens.GRCh37.dna.primary_assembly.fa] -I [xxx.bam] -O [xxx.bam]_output

put.g.vcf.gz -ERC GVCF --create-output-variant-index true --alleles [reich_autosomal_snp.vcf] -L [reich_autosomal_snp.vcf]

gatk CombineGVCFs -R [Homo_sapiens.GRCh37.dna.primary_assembly.fa] --disable-sequence-dictionary-validation true --lenient true \

-V [xxx.bam]__output.g.vcf.gz \

….. etc

-O [all.bam.combined]_GATK.g.vcf.gz

gatk --java-options "-Xmx4g" GenotypeGVCFs \

-R /[Homo_sapiens.GRCh37.dna.primary_assembly.fa] \

-V all.bam.combined]_GATK.g.vcf.gz \

-stand-call-conf 0 \

-O joint.all_call.vcf.gz

Pseudo-haploid random calls

A pipeline combining samtools mpileup and pileupCaller (ref) was used for pseudo-haploid random calls, required for READ and the Kennett method:

samtools mpileup **-R -B** -q30 -Q30 **-l** /workspaces/groups/adna-magdalenian/Universal/reich_autosomal_snp.txt \

**-f** /workspaces/groups/adna-magdalenian/Universal/reference/Homo_sapiens.GRCh37.dna.primary_assembly.fa \

**-b** **${NAME}** | **sort** -k1V | \

pileupCaller **--randomHaploid --sampleNameFile** ../**${TITLE}** \

**--samplePopName** **${NAME} -f** /workspaces/groups/adna-magdalenian/Universal/124*.snp \

**-e** ./1240k.**${NAME}**.convert.par.**${NAME}**.all

lcMLkin

lcMLkin uses genotype likelihood outputs from GATK, as above.

lcmlkin **-i** **${NAME}**/lcMLkin.input.vcf **-o** **${NAME}**/output.test.relate **-g** all **-t** 8 **-l** phred

NGSrelate

ANGSD was used as a dependency for NGSRelate to determine genotype likelihoods from a set of bam files. A minor allele frequency (MAF) of 0.05 was selected to overcome genotyping issues associated with the low coverage datasets.

angsd -b [xxx.bampathlist] -gl 2 -domajorminor 3 -sites [reich_autosomal_snp.txt] -domaf 1 -minmaf 0.05 -doGlf 3 -out [xxx.pathlist]

Output run in NGSRelate as below:

ngsRelate **-g** **${NAME}***.glf.gz **-n** 15 **-f** **${NAME}***_freq **-O** ./ngs_outputs/**${NAME}**.snp_ngsoutput **-z** short.name.list

READ

READ requires a plink file containing genotype calls. This was created using the PLINK –recode function and the output of pileupcaller as below:

Plink **--bfile** 1240k.**${NAME}**.convert.par.**${NAME}**.all **--recode --transpose --out** **${NAME}**/READ_plink

READ run as below (and thus using the mean normalisation method):

READ.py READ_plink

TKGWV2

TKGWV2 uses raw bam files, and a list of sites. In this instance, either the  22 million non-fixed biallelic sites (7) or the 1240k SNP panel (12,13) as mentioned previously.

TKGWV2.py bam2plink --referenceGenome /workspaces/groups/adna-magdalenian/Universal/reference/Homo*.fa --gwvList /workspaces/groups/adna-magdalenian/Universal/1000GP3_22M_noFixed_noChr.bed --bamExtension .bam --gwvPlink /workspaces/groups/adna-magdalenian/Universal/DummyDataset_EUR_22M_noFixed plink2tkrelate

d --freqFile /workspaces/groups/adna-magdalenian/Universal/1000GP3_EUR_1240K.frq - i 500

NGSRemix

NGSRemix requires input from the program ADMIXTURE, which itself requires a vcf file containing genotype information as created by the GATK (in the same manner as above).

plink **--vcf** **${NAME}**/joint.all_call.vcf.gz **--make-bed --out** **${NAME}**/NGSremix_plink_prefilter

plink **--bfile** **${NAME}**/NGSremix_plink_prefilter **--geno** 0.999 **--make-bed --out** **${NAME}**/NGSremix_plink

admixture **--cv** NGSremix_plink.bed **${NUMBER}** | tee log**${NUMBER}**.out

output was not only used to calculate the number of admixture elements present, but used as input for NGSremix:

INPUTPATH=$1

COVERAGE=$2

POPNO=$3

NGSremix -plink ${INPUTPATH}/${COVERAGE}/NGSremix_plink -f ${INPUTPATH}/${COVERAGE}/NGSremix_plink.${POPNO}.P -q ${INPUTPATH}/${COVERAGE}/NGSremix_plink.${

POPNO}.Q -P 2 -o ${INPUTPATH}/${COVERAGE}/${COVERAGE}.NGSremix.${POPNO}.output

Kennett et al. 2017

The method first described in Kennett et al 2017 requires genotype calls, which were produced as described above. All autosomal SNP from the 1240k SNP panel were used, with linkage sites being filtered prior to mismatch calculations (in PLINK). Pairwise mismatch rates were calculated using PLINK, and the normalisation formula implemented in base R.

plink **--bfile** 1240k.**${NAME}**.convert.par.**${NAME}**.all –-genome full

Using calculated mismatch rates, the relatedness coefficient ($\boldsymbol{r}$) was calculated using the formula below, where $\boldsymbol{x}$ is the pairwise mismatch rate and $\boldsymbol{b}$ equals half the maximum mismatch rate seen across all comparisons $\boldsymbol{b}: \frac{\max\left( \boldsymbol{x} \right)}{2}$.

$$\boldsymbol{r} : 1-\frac{(\boldsymbol{x}-\boldsymbol{b})}{\boldsymbol{b}}$$

**Kinship determination**

Both READ and TKGVW2 provide a determination of biological relatedness (first- or second-degree) as output that is calculated internally. To allow for the identification of any variance change in the associated kinship calculation across coverages, the normalized P0 value was extracted from the intermediate READ files and used as a proxy for the kinship coefficient ($\boldsymbol{\phi}$), with the HRC (half relationship coefficient) being extracted from the TKGVW2 output.

The output of the genotype likelihood methods (NGSrelate, lcMLkin and NGSremix) include the *k_0_, k_1_* and *k_2_* summary statistics that allowed for the calculation of the pairwise kinship coefficient ($\boldsymbol{\phi}$) using the following formula:

$$\boldsymbol{\phi} : \frac{\frac{k_{1}}{2}+k_{2}}{2}$$

These calculations provided a standardised value that allowed for the variation in kinship calculations between software methods and coverages to be determined.

**Determination of kinship variance across coverages and between packages**

The determination of variance was standardised within datasets and packages using the formula below, producing a Relative Kinship Score (RSK) for each pairwise comparison calculated:

$$\boldsymbol{RSK}_{\left( n in m \right)}: \frac{\left( \frac{\boldsymbol{\phi}_{\boldsymbol{n}}}{\max\left( \boldsymbol{\phi}_{\boldsymbol{m}} \right)} \right)}{\left( \frac{1}{\left( \frac{\boldsymbol{\phi}_{\boldsymbol{n}}}{\max\left( \boldsymbol{\phi}_{\boldsymbol{m}} \right)} \right)} \right)}$$

To standardise the proportion of total nucleotide sites used for each calculation both within each dataset and package, the following formula was used to calculate the proportion of the maximum number of pairwise sites (in dataset ***m***) for a specific pairwise relationship ***n***, where $\boldsymbol{x}$ is the number of nucleotide sites used for pairwise relationship:

$$\boldsymbol{Proportion of the maximum number of pairwise sites}_{\left( n in m \right)}: \frac{\left( \frac{\boldsymbol{x}_{\boldsymbol{n}}}{\max\left( \boldsymbol{x}_{\boldsymbol{m}} \right)} \right)}{\left( \frac{1}{\left( \frac{\boldsymbol{x}_{\boldsymbol{n}}}{\max\left( \boldsymbol{x}_{\boldsymbol{m}} \right)} \right)} \right)}$$

**Statistical Analysis**

The resultant plot of these two values can be seen in figure 1 of the main text and figures 1 and 2 of the supplementary information. Mantel tests on pairwise matrixes were carried out to identify any linear correlation between the relative kinship score and number of SNPs (where RKS is the Relative Kinship Score, and X is the proportion of max. pairwise sites for a specific pairwise relationship). Mantel tests were run using 250 permutations and random Monte Carlo sampling in R package vegan:

$$RKS \sim X$$

Calculations were carried out discretely for each dataset and software method, with the three ancient datasets being later combined and treated in a similar manner to the discrete datasets (*figure 1* of main text). Results for all pairwise calculations and mantel tests can be found in the accompanying supplementary tables.

**Supplementary Figures**

*****Figure S1:*** *The effect of low coverage data on biological relationship calculations using the R0, R1 and KING ratio method* (3)*. Colouration of datapoints signify the percentage of original reads sampled, and shape indicate the dataset each belong to. Comparative graphs showing the expected ratio distribution of a sample for specific biological relationships can be found in the source publication of NGSRelate* (5)

***
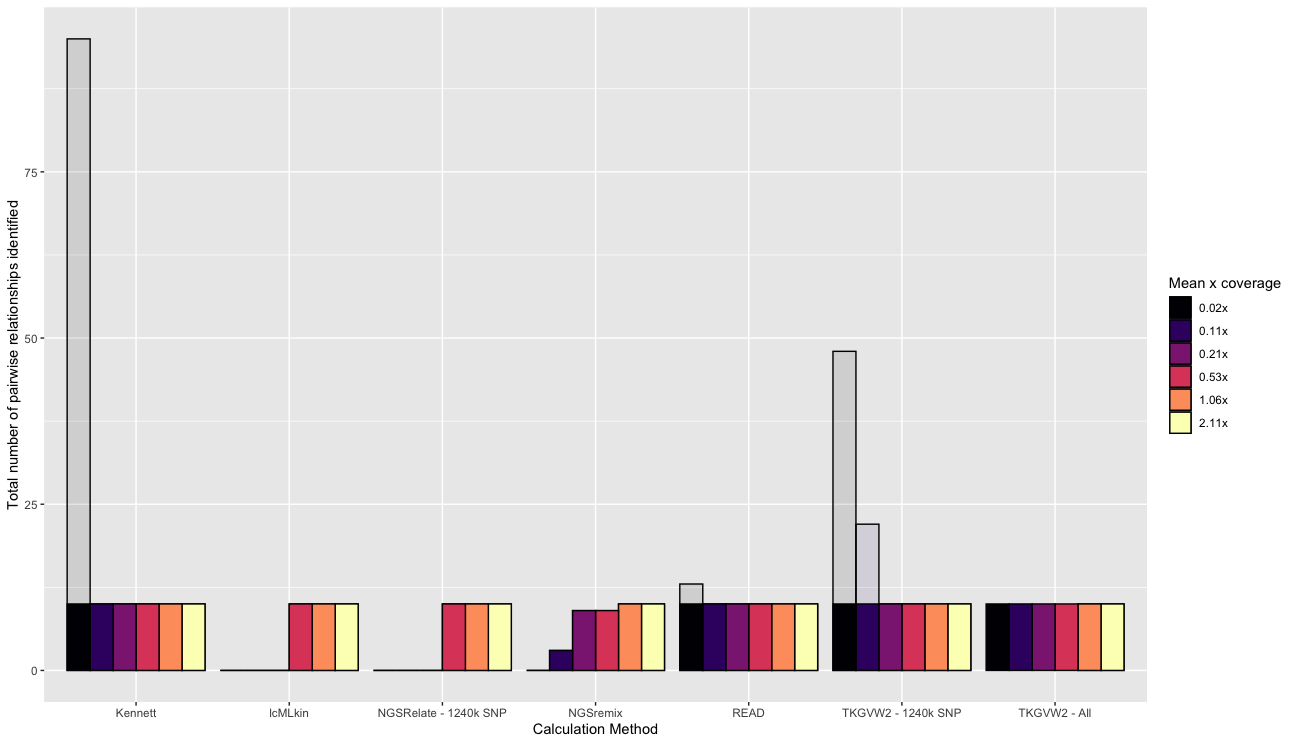
***

***Figure S2:*** *Variation in the number of pairwise relationships identified between packages and across coverages. Modern data used as input. Solid bars indicate the number of consistent relationships identified at maximum and reduced coverage (true positives). Translucent bars indicate the number of relationships across coverages not identified at maximum coverage (false positives).*

***
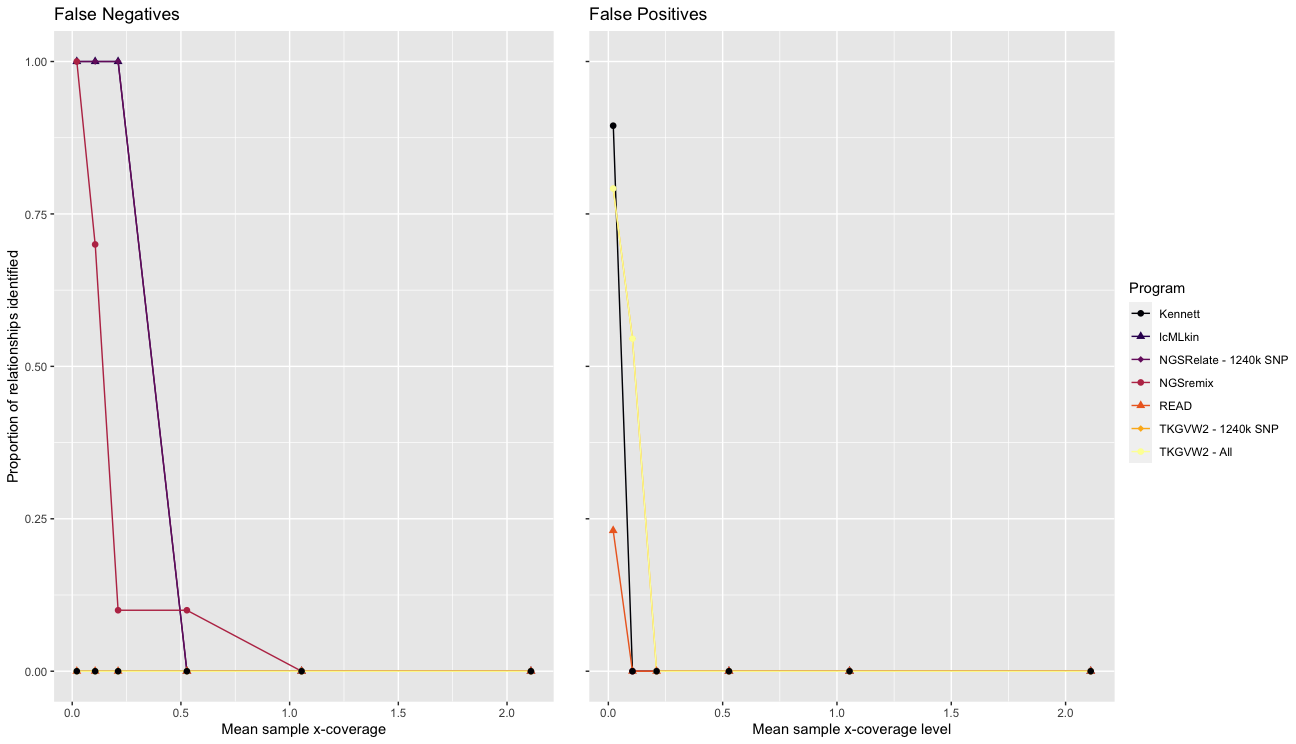
***

***Figure S3:*** *The consistency and accuracy (as defined in the methods) at all coverage levels for each kinship calculation method. Results from the modern dataset used as input (Table S1.10-14).*

**References:**

1. Schroeder H, Margaryan A, Szmyt M, Theulot B, Włodarczak P, Rasmussen S, et al. Unraveling ancestry, kinship, and violence in a Late Neolithic mass grave. Proc Natl Acad Sci U S A. 2019;166(22):10705–10.

2. Manichaikul A, Mychaleckyj JC, Rich SS, Daly K, Sale M, Chen WM. Robust relationship inference in genome-wide association studies. Bioinformatics. 2010;26(22):2867–73.

3. Waples RK, Albrechtsen A, Moltke I. Allele frequency-free inference of close familial relationships from genotypes or low-depth sequencing data. Mol Ecol. 2019;28(1):35–48.

4. Sikora M, Seguin-Orlando A, Sousa VC, Albrechtsen A, Korneliussen T, Ko A, et al. Ancient genomes show social and reproductive behavior of early Upper Paleolithic foragers. Science (80- ). 2017;358(6363):659–62.

5. Korneliussen TS, Moltke I. NgsRelate: A software tool for estimating pairwise relatedness from next-generation sequencing data. Bioinformatics. 2015; 31(24) 4009-4011.

6. Margaryan A, Lawson DJ, Sikora M, Racimo F, Rasmussen S, Moltke I, et al. Population genomics of the Viking world. Nature. 2020;585(7825):390–6.

7. Auton A, Abecasis GR, Altshuler DM, Durbin RM, Bentley DR, Chakravarti A, et al. A global reference for human genetic variation. Nature. 2015;526(7571):68–74.

8. Margaryan A, Lawson D, Sikora M, Racimo F, Rasmussen S, Moltke I, et al. Population genomics of the Viking world. bioRxiv. 2019; 585:703405. Available from: http://dx.doi.org/10.1038/s41586-020-2688-8

9. Schubert M, Lindgreen S, Orlando L. AdapterRemoval v2: Rapid adapter trimming, identification, and read merging. BMC Res Notes. 2016;9(1):1–7.

10. Li H, Durbin R. Fast and accurate short read alignment with Burrows-Wheeler transform. Bioinformatics. 2009; 25(14):1754-1760.

11. Li H, Handsaker B, Wysoker A, Fennell T, Ruan J, Homer N, et al. The Sequence Alignment/Map format and SAMtools. Bioinformatics. 2009; 25(16):2078-2079.

12. Lazaridis I, Patterson N, Mittnik A, Renaud G, Mallick S, Kirsanow K, et al. Ancient human genomes suggest three ancestral populations for present-day Europeans. Nature [Internet]. 2014;513(7518):409–13. Available from: http://dx.doi.org/10.1038/nature13673

13. Mathieson I, Lazaridis I, Rohland N, Mallick S, Patterson N, Roodenberg SA, et al. Genome-wide patterns of selection in 230 ancient Eurasians. Nature. 2015;528(7583):499–503.
